# Systematic analysis of gene expression in human brains before and after death

**DOI:** 10.1186/gb-2005-6-13-r112

**Published:** 2005-12-30

**Authors:** Henriette Franz, Claudia Ullmann, Albert Becker, Margaret Ryan, Sabine Bahn, Thomas Arendt, Matthias Simon, Svante Pääbo, Philipp Khaitovich

**Affiliations:** 1 Max-Planck-Institute for Evolutionary Anthropology, Deutscher Platz, D-04103 Leipzig, Germany; 2 Department of Neuropathology and National Brain Tumor Reference Center, University of Bonn Medical Center, Sigmund-Freud-Strasse, D-53105 Bonn, Germany; 3 Cambridge Centre for Neuropsychiatric Research, Institute of Biotechnology, University of Cambridge, Tennis Court Road, Cambridge CB2 1QT, UK; 4 Paul Flechsig Institute for Brain Research, University of Leipzig, Jahnallee, D-04109 Leipzig, Germany; 5 Department of Neurosurgery, University of Bonn Medical Center, Sigmund-Freud-Strasse, D-53105 Bonn, Germany

## Abstract

**Background:**

Numerous studies have employed microarray techniques to study changes in gene expression in connection with human disease, aging and evolution. The vast majority of human samples available for research are obtained from deceased individuals. This raises questions about how well gene expression patterns in such samples reflect those of living individuals.

**Results:**

Here, we compare gene expression patterns in two human brain regions in postmortem samples and in material collected during surgical intervention. We find that death induces significant expression changes in more than 10% of all expressed genes. These changes are non-randomly distributed with respect to their function. Moreover, we observe similar expression changes due to death in two distinct brain regions. Consequently, the pattern of gene expression differences between the two brain regions is largely unaffected by death, although the magnitude of differences is reduced by 50% in postmortem samples. Furthermore, death-induced changes do not contribute significantly to gene expression variation among postmortem human brain samples.

**Conclusion:**

We conclude that postmortem human brain samples are suitable for investigating gene expression patterns in humans, but that caution is warranted in interpreting results for individual genes.

## Abstract 2

Comparison of the gene expression profiles of pre- and post-mortem human brains suggests that post-mortem human brain samples are suitable for investigating general gene-expression patterns.

## Background

Microarray studies examining gene expression profiles of thousands of genes have become an important tool in uncovering molecular mechanisms of human diseases, aging and evolution [1-3]. Many such studies are conducted on postmortem human tissues, since neither cell culture nor animal models can fully recapitulate relevant human conditions [4,5]. This is particularly the case for studies that examine the human brain. Several factors may alter gene expression profiles in postmortem human brain samples. Such factors include the delay between death and the time of tissue freezing, the method of freezing, and the duration of storage of the frozen brain material. Prior studies have indicated that these factors have relatively small effects on gene expression [6-8]. In contrast, the duration and nature of the agonal state preceding death appear to have a substantial effect on gene expression by affecting the integrity of messenger RNAs [7-9]. Thus, postmortem brain samples obtained from individuals who died after a protracted agonal phase are not suitable for gene expression studies. Without any prolonged agonal conditions, however, death itself may alter gene expression patterns in postmortem human brains. Study of expression levels of 14 genes in human brain autopsy and biopsy samples found significant change in one of the genes, indicating that a substantial proportion of all expressed genes could be affected by death [10].

We surveyed gene expression in 10 postmortem human brain samples (autopsy samples) and 12 samples obtained from brain surgery (resection samples) derived from frontal cortex and hippocampus using Affymetrix^® ^HG-U133plus2 microarrays containing probes for all annotated human genes. All autopsy samples were obtained from individuals that died rapidly with no prolonged agonal state, thus minimizing the influence of agonal factors on gene expression patterns in our study.

## Results

### Expression differences between autopsy and resection samples

**Figure 1:**
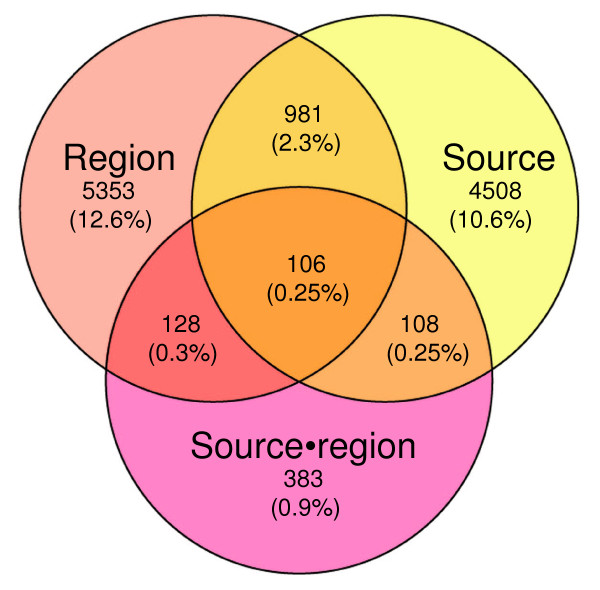
ANOVA test results. Numbers indicate number of probe sets with expression significantly influenced by brain region, source of sample material, and their interaction. The interaction term is significant when the expression changes due to death differ significantly in the two brain regions examined (see Material and methods). Numbers in brackets indicate the percentage of significant probe sets compared to the total number included in the analysis. Overlapping regions include probe sets with more than one significant term.

Gene expression profiles were determined in six resection samples from hippocampus and frontal cortex, and in four and six autopsy samples from hippocampus and frontal cortex, respectively, using Affymetrix^® ^HG U133plus2 arrays (see Materials and methods). Of the 54,613 probe sets on the microarray, 42,427 (77.69%) gave a detectable hybridization signal in at least one individual (see Materials and methods). Among these probe sets, we found 5,703 with a significant difference in expression (13.4%) using analysis of variance (ANOVA) with a nominal significance cutoff of 0.01 (false discovery rate (FDR) = 4.12%, permutation test) and 8,643 using significance analysis of microarrays (SAM) at the 5% FDR cutoff. Out of the 5,703 probe sets identified in ANOVA, 5,515 (96.7%) overlapped with the probe sets identified by SAM. Further, of these 5,703 probe sets, 4,508 differed significantly (*p *< 0.01) between autopsy and resection samples in both brain regions while 981 probe sets showed a significant difference between autopsy and resection samples as well as between brain regions (Figure 1). For none of these 5,489 probe sets did the differences between autopsy and resection samples depend significantly on the brain region. Finally, for 214 probe sets (0.5% of all detected ones), expression differences between autopsy and resection samples differed significantly (*p *< 0.01) depending on the brain region examined. This indicates that death-induced expression changes are highly consistent in both brain regions and influence only a small fraction of the total observed expression differences (214 out of 5,703).

Since all but one surgery patient were diagnosed with epilepsy (Table 1), we first tested whether differences between autopsy and resection samples are significantly affected by the epileptic condition. Among the 42,427 expressed probe sets, we found none with a significant effect of epilepsy either in hippocampus or in frontal cortex using both linear regression and SAM (FDR = 5.0%). Further, we tested whether known changes in expression caused by epilepsy are over-represented among differences seen between autopsy and resection samples. Using a published set of genes where expression change was observed in at least two epilepsy studies (*N *= 54) [11], we found no such over-representation (Fisher's exact test, *p *= 0.45). Finally, we tested whether expression differences we found between autopsy and resection are also seen when only the samples unaffected by epilepsy are considered. To this end, we identified probe sets showing expression differences between autopsy and resection samples, excluding from the analysis samples from patients not affected by epilepsy (ANOVA, *p *< 0.01). We found a strong and significant correlation when these expression differences were compared to the ones observed in non-affected control samples; three resections composed of two cerebral cortex samples from an unaffected region and one hippocampus sample from a non-epileptic patient gave Pearson's correlation *R *= 0.948 (*N *= 2,983, *p *< 10^-15^) or using the one hippocampus sample only gave Pearson's correlation *R *= 0.905 (*N *= 4,088, *p *< 10^-15^). Thus, the overwhelming majority of expression differences between autopsy and resection identified in samples affected by epileptic condition are also present in the non-affected samples.

We next asked whether the genes represented by the 4,508 probe sets that showed significant differences in expression between autopsy and resection samples in both brain regions cluster in functional categories as defined by the Gene Ontology (GO) consortium [12]. Differently expressed genes clustered significantly in all three GO taxonomies, 'biological process', 'molecular function' and 'cellular component' (*p *< 0.0001). Among 15 GO 'biological process' categories with significant over-representation of differently expressed genes, four are involved in cellular protein metabolism and six in nucleobase, nucleoside, nucleotide and nucleic acid metabolism. Most of the remaining genes are found in the categories 'organelle organization and biogenesis' and 'intracellular protein transport' (Table 2). The expression of genes involved in the ubiquitin cycle and protein ubiquitination is significantly increased after death, while the expression of genes involved in protein biosynthesis, rRNA processing, organelle organization and biogenesis and induction of apoptosis are significantly decreased (two-sided binomial test, *p *< 0.05).

Among 20 GO categories with significant under-representation of genes differently expressed between autopsies and resections, seven are involved in cell communication, three in response to stimulus, two in sensory perception, and four in development. In addition, 'cellular physiological process' and 'organismal physiological process' are among the GO categories that are significantly conserved in their expression between autopsy and resection samples (Table 2).

In contrast, no chromosome showed either an excess or lack of expression differences (two-sided binomial test, *p * < 0.341, corrected for multiple testing).

### Expression differences between brain regions

To test whether *in vivo *expression differences between the brain regions are conserved in postmortem samples, we first considered the ANOVA results (Figure 1). Among 42,427 probe sets with hybridization signals detectable in at least one individual, 6,568 (15.5%) showed significant expression differences between the two brain regions analyzed (nominal significance *p *< 0.01, FDR = 3.6%, permutation test). Out of these probe sets, 6,431 (97.9%) overlapped with the ones identified by SAM (FDR = 5%). In 234 of these 6,431 probe sets, differences between brain regions depended significantly on the source of sample material (*p *< 0.01). Thus, although autopsy and resection samples differ substantially with regard to their gene expression profiles, the patterns of expression differences between the brain regions remain largely preserved.

**Figure 2:**
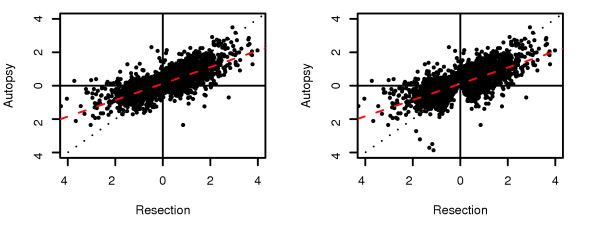
Scatter plot of expression differences between cortex and hippocampus in resection (x-axis) and autopsy (y-axis) samples. Expression differences were calculated as base two logarithm transformed ratios of gene expression values. All probe sets showing significant differences in expression levels between the two brain regions, either in the autopsy or in resection samples, are plotted: **(a) **according to Student's *t* test; **(b) **according to SAM. Red dashed lines represent linear regression results and black dotted lines represent expected regression lines with the slope = 1.

We tested further whether *in vivo *expression differences between the brain regions are conserved in the postmortem samples by separately identifying, independent of the ANOVA results, probe sets differently expressed between the brain regions in the autopsy and in the resection samples. Using Student's *t* test with nominal significance *p *< 0.01, we found 788 and 3,943 probe sets with a significant difference in expression between the brain regions in the autopsy and in the resection samples, respectively (FDR = 22.8% and 4.3% respectively, permutation test). Similarly, using SAM with FDR = 5% we found 874 and 6,699 probe sets with a significant difference in expression between the brain regions in the autopsy and in the resection samples, respectively. This large discrepancy in the numbers of differences between the brain regions when the autopsy and resection samples are considered separately seems to contradict the ANOVA results. To address this, we examined whether probe sets that do not overlap between these two lists tend to show the same pattern of change between the brain regions or, alternatively, are completely uncorrelated in their expression behavior. For this purpose, we considered all probe sets present on either of the two lists and calculated the strength of correlation of the expression difference between the brain regions measured in the autopsy and in the resection samples. We found a strong and significant correlation between the expression differences for both *t* test (Pearson's correlation *R *= 0.763, *N *= 4,471, *p *< 10^-15^) and SAM results (Pearson's correlation *R *= 0.726, *N *= 7,162, *p *< 10^-15^) (Figure 2). Similarly, we found slightly reduced but still highly significant correlations using expression differences normalized to the average variation (effect size) (Pearson's correlation *R *= 0.566, *p *< 10^-15 ^and *R *= 0.584, *p *< 10^-15^, respectively). Thus, expression differences betweenthe two brain regions are largely concordant in the autopsy and resection samples. Interestingly, the slopes of the regression lines (*β*) fitted through the distributions of the expression differences between the two brain regions in the autopsy and the resection samples equal 0.49 for both sets of genes (Figure 2). An even stronger effect was observed using the effect size measurements (*β *= 0.33 and *β *= 0.32 for *t* test and SAM results, respectively). Thus, despite an overall agreement of the measurements of expression differences in the two sources of sample material, the amplitude of expression differences measured in the autopsy samples is, on average, half of that observed in the resection samples. Limiting the regression to genes with a high expression difference amplitude in either autopsy or resection samples did not change this effect. Interestingly, it was even more pronounced for genes with lower expression in the frontal cortex compared to the hippocampus (*β *= 0.27 and *β *= 0.34 for *t* test and SAM results, respectively). Since the significance test depends on the effect size, smaller expression differences explain the reduced number of identified probe sets in the autopsy samples.

### Influence of death on expression variation

All microarray studies involving postmortem human samples report substantial biological variation among individuals. We asked whether death-induced expression changes contribute to this variation by affecting different individuals to different degrees. To do this, we examined published gene expression data from 40 brain autopsy samples [13]. First, we asked whether probe sets that differ in expression between autopsy and resection samples vary more among individuals in this dataset than other probe sets. From the 16,376 probe sets with a detectable hybridization signal in at least one of the 40 individuals, 1,752 overlap with the probe sets showing significant differences in expression between autopsy and resection samples. Using logarithm transformed variation measures, we found no significant difference between the expression variation among these probe sets and among the remaining probe sets (Student's *t* test, *p *= 0.916). Thus, genes that differ in expression between autopsy and resection samples do not vary more among postmortem samples compared to the other genes.

Next, we asked whether the amplitude of death-induced expression changes correlates with the duration of postmortem interval. To test this, we computed correlations between gene expression levels and postmortem delay in the 40 brain autopsy samples for 1,752 probe sets that differ in expression between autopsy and resection samples and for 1,000 subsets of the same size randomly sampled from the other 14,624 probe sets. In 837 out of 1,000 random subsets, the correlation was greater or equal to the one observed for probe sets with significant difference in expression between autopsy and resection samples. Thus, genes that differ in expression between autopsy and resection samples do not correlate more with duration of postmortem interval than the rest of the detected genes.

## Discussion

In this study, we observe that death causes substantial changes in the expression of more than 10% of genes expressed in human brain. Furthermore, this change is highly reproducible, with 96% of differences being shared when two very different brain regions (frontal cortex and hippocampus) are considered. Since all brain resection samples were obtained from people with certain brain abnormalities, an alternative explanation is that the observed changes are induced by disease of the living brain rather than by death. However, for several reasons we find this explanation unlikely. First, we used resection samples from patients suffering from several different neurological disorders (Table 1), which are not likely to induce the same pattern of gene expression change. Second, although all but one of the patients were diagnosed with epilepsy, severity of the disease did not significantly influence expression differences between autopsy and resection samples. Third, we observed similar gene expression differences between autopsy and resection samples in both frontal cortex and hippocampus. It is unlikely that these brain regions are affected in the same way by the diseases in question. Finally, we found consistent gene expression differences in the four frontal cortex samples affected by disease at the histological level and the ones with normal histology. Taken together, these arguments suggest that the gene expression differences we observed between autopsy and resection samples are not due to disease-induced change in the resection samples.

Still, two factors, epilepsy and surgery, are shared among most or all patients, respectively. We found no genes with a significant effect of epilepsy on expression either in hippocampus or in frontal cortex. Similarly, using data from the resection samples of non-epileptic patients, we found the same expression differences between autopsy and resection samples as we found with epileptic patients' samples. In addition, known expression changes induced by epilepsy are not over-represented among differences between autopsy and resection samples. These results indicate that epilepsy is unlikely to have contributed a great deal to the expression differences we see. Due to the small number of samples used in the analysis, however, we cannot completely exclude such an effect. Similarly, we cannot exclude influence of surgery and surgery related treatments, like anesthesia, on gene expression in all resection samples. This remains a confounding factor for estimation of the expression differences between postmortem and living human brain tissue that we cannot address in this study.

Yet, given the widespread use of postmortem human brain tissue in research, the most important question is how well gene expression differences measured in postmortem samples reflect those occurring *in vivo*. We found that despite the large impact that death as such and, potentially, surgery have on gene expression patterns in autopsy and resection samples, respectively, differences between brain regions that exist in the living brain are mostly retained in postmortem samples. However, it is striking that the magnitude of the expression differences between the two brain regions decreases by approximately 50% on average and that the effect size is reduced by approximately two-thirds in postmortem samples. This reduction did not depend on the magnitude of difference. Interestingly, the reduction was even more pronounced in genes with lower expression in frontal cortex than in hippocampus (Figure 2). This indicates that gene expression differences measured in postmortem brain samples may underestimate differences existing in the living tissue.

Interestingly, gene expression changes induced by death do not appear to increase variation among postmortem brain samples. In agreement with this, we found no significant correlation between the duration of postmortem interval and the magnitude of expression differences between autopsy and postmortem samples. This suggests that expression changes occur quickly in the process of dying and remain stable thereafter. This observation is in agreement with recent findings that postmortem delay does not substantially influence gene expression variation among human brain samples [6-8], whereas prolonged agonal states significantly influence expression profiles.

The genes that differ in their expression between autopsy and resection samples are significantly over- and under-represented in certain functional processes. Genes involved in rather basic functions, such as RNA processing, protein biosynthesis and transport, organelle organization and biogenesis, the ubiquitin cycle, and DNA repair (Table 1) are over-represented among genes differently expressed between autopsies and resections. We would have expected an overall down-regulation of these pathways in tissues after death. Indeed, genes involved in rRNA processing, protein biosynthesis, induction of apoptosis, and organelle organization and biogenesis show significant down-regulation in the autopsy samples. Interestingly, we also see up-regulation of genes involved in the ubiquitin cycle, protein ubiquitination, and ubiquitin-dependent protein catabolism. This implies that death leads to the temporary induction of expression for some functional processes. It is intriguing to think that death does not lead to immediate shut down of all functional processes on a cellular level. If these transcripts become translated to functional proteins, up-regulation of genes involved in ubiquitin-dependent protein catabolism may lead to increased degradation of proteins in human brain samples after death. This could have consequences for protein studies in postmortem human brain samples, where protein degradation is commonly observed [14-16]. It may thus be important to compare protein patterns in postmortem andresection samples of human brains to estimate the extent of death-induced protein degradation.

More than three quarters of the GO categories with significant conservation of their expression levels after death fall into processes involved in intra- and extracellular signaling and in development (Table 1). This is rather unexpected since these processes underlie essential brain functions and genes involved in such functions have been shown to differ in their expression levels among various brain regions [17,18]. Intuitively, one might expect that death would affect these processes first. The excess or paucity of expression differences in certain functional processes could be caused by differences in RNA degradation rates. In this case we would expect genes with low RNA turnover to fall into functional categories that maintain their observed expression levels after death and genes with high RNA turnover to fall into significantly changed functional categories. However, genes involved in signal transduction and development are known to have high RNA turnover rates [19,20] while genes involved in general metabolic functions, biosynthesis and catabolism have low RNA turnover rates [20,21]. Thus, it is unlikely that the observed clustering of expression differences in distinct functional categories is due to differences in RNA degradation rates.

## Conclusion

Despite the large effect of death on gene expression in human brain, postmortem samples maintain the vast majority of the expression differences that exist between brain regions *in vivo*. However, the amplitude of expression differences between brain regions in postmortem samples is reduced by approximately 50% compared to the living tissue. It should be noted that the results reported here examined only a limited number of samples representing only few conditions and that confounding effects, including surgery and anesthesia, may influence some of the expression differences we observe. Nevertheless, given that the primary source of brain tissue is postmortem collection, it is encouraging that there is such a high degree of correlation in gene expression patterns between sources.

## Materials and methods

### Tissue samples and microarray data collection

Human postmortem samples were obtained from the National Disease Research Interchange. Informed consent for use of the tissues for research was obtained in writing from all donors or the next of kin. None of the subjects had a history of neurological disease or had indications of brain abnormalities at the tissue level as determined at autopsy. All individuals suffered sudden death for reasons other than their participation in this study and without any relation to the tissues used. Human resection samples were obtained from patients with brain tumors and/or chronic pharmaco-resistant epilepsy who underwent surgical treatment in the Surgery/Epilepsy Surgery Programs at the University of Bonn Medical Center. In all patients, surgical removal of the tumor/lesion tissue was necessary. Informed consent for additional studies was obtained in writing from all patients. The diagnosis of the individual patients is presented in Table 1. All procedures were conducted in accordance with the Declaration of Helsinki and approved by the ethics committees of the respective institutions. Representative tissue sections were snap frozen at -80°C. Based on neuropathological analyses by means of hematoxilin and eosin stainings, normal tissue adjacent to the tumor or lesions was used for subsequent experiments. Intense care was taken to avoid tumor infiltrated tissue. None of the surgically obtained tissue samples used in this study, with the exception of four frontal cortex samples with focal cortical dysplasia, showed any histological abnormalities. Age, sex, and degree of relatedness of all individuals are listed in Table 1. 

All samples were processed in parallel starting from the frozen tissue by the same person (HF) in random order with respect to brain region and the source of sample material. Total RNA was isolated from approximately 50 mg of frozen tissue using TRIZol^® ^(GIBCO, San Diego, CA, USA) reagent according to the manufacturer's instructions and purified with QIAGEN^® ^RNeasy^® ^kit (Valencis, CA, USA) following the 'RNA cleanup' protocol. All RNA samples were of high and comparable quality as determined by the ratio of 28S to 18S ribosomal RNAs estimated using the Agilent^® ^(Palo Alto, CA, USA) 2100 Bioanalyser^® ^system and by the signal ratios between the probes for the 5' and 3' ends of the mRNAs of GAPDH used as quality controls on Affymetrix^® ^(Santa Clara, CA< USA) microarrays (Table 1). Labeling of 1.2 μg of total RNA, hybridization to Affymetrix^® ^HG U133plus2 arrays, staining, washing and array scanning were carried out following Affymetrix^® ^protocols. All primary expression data are publicly available at the ArrayExpress database (accession number E-TABM-20) [22].

### Microarray data analyses

Affymetrix^® ^microarray image data were collected with Affymetrix^® ^GeneChip^® ^Operating Software version 1.1 using default parameters. We used the robust multichip average (rma) procedure [23] for array normalization and calculation of expression base two logarithm transformed intensity values. Since logarithm-transformed intensity values are approximately normally distributed, we used them for all analyses. We calculated detection *p *values using the Bioconductor 'affy' software package [24]. We defined probe sets having a detectable hybridization signal using Affymetrix default detection cutoff of 0.065.

We used ANOVA to identify probe sets that showed a statistically significant change in expression depending on the brain region or on the source of sample material among human samples using the following model: *Y*_*ij *_= μ_*j *_+ source_*i *_+ region_*i *_+ (source*region)_*i *_+ ε_*ij*_. In this equation, Y_*ij *_is the base two logarithm of the expression level for probe set *j *in sample *i*, μ is the mean expression level of a probe set *j*, source_*i *_is the term for the effect of the source of sample material, region_*i *_is the term for the effect of the source of the brain region, (source*region)_*i *_is the term for the interaction effect of the two factors, and ε_*ij *_is the error term. For each term we used a nominal significance level of 0.01. In order to estimate an average number of probe sets expected by chance at this significance cutoff, we applied the same ANOVA approach to 1,000 datasets constructed by random permutation of the sample labels in the original data.

Alternatively, differently expressed probe sets were determined using SAM software version 2.01 with 5% FDR cutoff [25]. In all cases except the analysis of epilepsy effects, we performed *t* statistics on the logarithm transformed expression values. FDR estimates were based on 500 permutations of the samples within the set. We used block permutation design for the two-factor analysis and time course for the analysis of epilepsy effects. Effect of epilepsy was scored based on the diagnosis and seizure type: 0, no diagnosed epilepsy; 1, simple partial seizures; 2, simple and complex partial seizures; 3, complex partial seizures; 4, simple and complex partial seizures, grand mal; 5, complex partial seizures. Effect size was calculated as a difference between means divided by the pooled standard deviation. The pooled standard deviation was defined as the square root of the average of the squared standard deviations.

### Functional analysis and distribution on chromosomes

To functionally annotate the probe sets on the Affymetrix^® ^HG U133plus2 arrays, we integrated information from four public databases: Affymetrix^® ^NetAffx™ (12/2004 release) [26], LocusLink (12/2004 release) [27], and Gene Ontology (12/2004 release) [28]. Affymetrix^® ^probe sets were linked to the corresponding genes using LocusLink annotation provided by NetAffx™. When a single gene was represented by multiple probe sets, the gene was classified as detected if at least one probe set was detected and classified as differentially expressed if at least one probe set was both detected and differentially expressed. Genes were assigned to their GO annotations from each of the three GO taxonomies ('molecular function', 'biological process', and 'cellular component') using GenMapper [29,30]. Note that a gene belongs to its assigned GO group as well as all higher groups in the taxonomy.

To assess if the overall distribution of genes differentially expressed between autopsy and resection samples across the groups in a GO taxonomy differs significantly from the distribution of all detected genes, we compared it with 10,000 random sets in which the same number of differentially expressed genes was randomly drawn from the annotated detected genes as described elsewhere [18]. GO groups with significant excess and with significant lack of expression differences between autopsy and resection samples were determined independently using the hypergeometric distribution [18]. The percentage of false positive GO groups was estimated from the ratio of the number of significant groups in the observed data to the average number of the significant groups in 10,000 random sets. In the GO taxonomy 'biological process', we expect 20% false positives for the groups with significant excess and 5.8% false positives for the groups with significant lack of expression differences between autopsy and resection samples. Significant over-representation of up- or down-regulated genes in GO groups with significant excess of expression differences was determined by binomial test. Probability of up- and down-regulation within a group was based on distribution of all differently expressed genes. To assign chromosomal location to genes we used annotation provided by NetAffx™. Genes differently expressed between autopsy and resection samples were defined the same way as for the functional analysis.

## Figures and Tables

**Table 1:** Sample information

Sample*	Age (years)	Sex	28S/18S ratio^†^	GAPDH 5'/3' ratio^‡^	Expressed probe sets (%)^§^	Diagnosis	Epilepsy	Types of seizures
HA1	70	M	1.2	0.445	50.6	-	-	-
HA3	45	M	1.6	0.637	49.7	-	-	-
HA4	45	M	1.2	0.507	49.4	-	-	-
HA5	54	F	1.6	0.712	51.7	-	-	-
HR1	45	M	1.1	0.520	50.5	Anaplastisches Oligo WHO III	Yes	Simple partial
HR2	39	F	1.3	0.700	50.2	Glioblastoma	Yes	Simple and complex partial, GM
HR3	61	M	1.6	0.774	53.8	Glioblastoma	Yes	Simple and complex partial
HR4	51	F	1.6	0.697	49.5	Ammon's horn sclerosis	Yes	Simple and complex partial, GM
HR5	13	M	1.4	0.778	47.1	Ganglioglioma	Yes	Complex partial
HR6	83	F	1.3	0.817	50.0	Atpisches Meningeom Grad II	No	-
CA1	45	M	1.4	0.870	51.0	-	-	-
CA2	45	M	1.4	0.841	51.4	-	-	-
CA3	48	M	1.5	0.865	53.2	-	-	-
CA5	70	M	1.4	0.669	47.2	-	-	-
CA6	82	F	1.7	0.690	47.7	-	-	-
CA7	67	M	NA	0.810	49.5	-	-	-
CR1	35	F	1.2	0.741	45.9	Focal cortical dysplasia	Yes	Complex partial, GM
CR2	31	F	1.3	0.741	39.5	Focal cortical dysplasia	Yes	Simple partial
CR3	9	F	NA	0.607	45.6	Focal cortical dysplasia	Yes	Complex partial
CR4	37	M	NA	0.674	43.7	Focal cortical dysplasia	Yes	Complex partial
CR5	35	F	NA	0.737	48.8	Focal cortical dysplasia	Yes	Complex partial, GM
CR6	31	F	NA	0.674	43.1	Focal cortical dysplasia	Yes	Simple partial

*Sample names: position one = brain region (H, hippocampus; C, cortex); position two = sample source (A, autopsy; R, resection); position three = individual. ^†^Ribosomal RNA bands ratio was measured using Agilent 2100 Bionalyzer system. ^‡^GAPDH ratio was measured using probes to 5' and 3' of the transcript on Affymetrix^® ^array. ^§^Expressed probesets were defined based on detection *p *< 0.05. F, female; GM, grand mal; M, male; NA, not applicable.

**Table 2:** Functional analysis of gene expression differences between autopsy and resection samples

GO ID	Term	Expressed genes	Significant differences*	Change *p *value	Conservation *p *value
GO:0006412	Protein biosynthesis	462	101 (**37/64**)	0.001	0.999
GO:0006512	Ubiquitin cycle	473	119 (**86/33**)	0.000	1.000
GO:0016567	Protein ubiquitination	256	60 (**41/19**)	0.002	0.999
GO:0006511	Ubiquitin-dependent protein catabolism	104	36 (23/13)	0.000	1.000
GO:0006396	RNA processing	341	118 (64/54)	0.011	0.995
GO:0006397	mRNA processing	217	74 (44/30)	0.002	0.999
GO:0008380	RNA splicing	183	67 (39/28)	0.000	1.000
GO:0006281	DNA repair	168	40 (23/17)	0.009	0.995
GO:0000398	Nuclear mRNA splicing, via spliceosome	155	54 (30/24)	0.000	1.000
GO:0006364	rRNA processing	32	16 (**3/13**)	0.000	1.000
GO:0006996	Organelle organization and biogenesis	367	83 (**30/53**)	0.048	0.964
GO:0006886	Intracellular protein transport	263	62 (32/30)	0.002	0.999
GO:0008624	Induction of apoptosis by extracellular signals	28	13 (**2/11**)	0.000	1.000
GO:0006120	Electron transport, NADH to ubiquinone	24	10 (3/7)	0.003	0.999
GO:0048247	Lymphocyte chemotaxis	3	3 (0/3)	0.004	1.000
GO:0007242	Intracellular signaling cascade	879	105	0.989	0.016
GO:0007186	GPCR protein signaling pathway	448	39	1.000	0.000
GO:0007267	Cell-cell signaling	417	39	0.998	0.003
GO:0007243	Protein kinase cascade	231	24	0.997	0.005
GO:0045860	Positive regulation of protein kinase activity	41	1	0.999	0.006
GO:0007268	Synaptic transmission	203	18	0.999	0.001
GO:0007187	G-protein signaling (cyclic nucleotide second messenger)	73	4	0.999	0.004
GO:0050896	Response to stimulus	1,326	179	0.975	0.035
GO:0009605	Response to external stimulus	781	90	0.972	0.037
GO:0009617	Response to bacteria	37	0	1.000	0.001
GO:0007601	Visual perception	126	9	0.999	0.002
GO:0007606	Sensory perception of chemical stimulus	55	2	0.999	0.003
GO:0007275	Development	1,412	174	0.992	0.011
GO:0009887	Organogenesis	770	89	0.997	0.004
GO:0007417	Central nervous system development	92	6	0.999	0.004
GO:0008544	Epidermis development	39	1	0.999	0.008
GO:0050875	Cellular physiological process	3,372	515	1.000	0.000
GO:0050874	Organismal physiological process	1,200	138	0.997	0.004
GO:0006813	Potassium ion transport	139	3	1.000	0.000
GO:0030003	Cation homeostasis	52	1	1.000	0.001

*Numbers in parenthesis correspond to the number of up- and down-regulated genes in the autopsy samples. Bold font indicates Gene Ontology (GO) groups with significant excess of up- or down-regulated genes (see Materials and methods).

## References

[B1] Marcotte ER, Srivastava LK, Quirion R (2003). cDNA microarray and proteomic approaches in the study of brain diseases: focus on schizophrenia and Alzheimer's disease.. Pharmacol Ther.

[B2] Preuss TM, Caceres M, Oldham MC, Geschwind DH (2004). Human brain evolution: insights from microarrays.. Nat Rev Genet.

[B3] Vijg J, Calder RB (2004). Transcripts of aging.. Trends Genet.

[B4] Gotz J, Streffer JR, David D, Schild A, Hoerndli F, Pennanen L, Kurosinski P, Chen F (2004). Transgenic animal models of Alzheimer's disease and related disorders: histopathology, behavior and therapy.. Mol Psychiatry.

[B5] Soutourina O, Cheval L, Doucet A (2005). Global analysis of gene expression in mammalian kidney.. Pflugers Arch.

[B6] Bahn S, Augood SJ, Ryan M, Standaert DG, Starkey M, Emson PC (2001). Gene expression profiling in the post-mortem human brain - no cause for dismay.. J Chem Neuroanat.

[B7] Li JZ, Vawter MP, Walsh DM, Tomita H, Evans SJ, Choudary PV, Lopez JF, Avelar A, Shokoohi V, Chung T (2004). Systematic changes in gene expression in postmortem human brains associated with tissue pH and terminal medical conditions.. Hum Mol Genet.

[B8] Tomita H, Vawter MP, Walsh DM, Evans SJ, Choudary PV, Li J, Overman KM, Atz ME, Myers RM, Jones EG (2004). Effect of agonal and postmortem factors on gene expression profile: quality control in microarray analyses of postmortem human brain.. Biol Psychiatry.

[B9] Hynd MR, Lewohl JM, Scott HL, Dodd PR (2003). Biochemical and molecular studies using human autopsy brain tissue.. J Neurochem.

[B10] Castensson A, Emilsson L, Preece P, Jazin EE (2000). High-resolution quantification of specific mRNA levels in human brain autopsies and biopsies.. Genome Res.

[B11] Lukasiuk K, Pitkanen A (2004). Large-scale analysis of gene expression in epilepsy research: is synthesis already possible?. Neurochem Res.

[B12] Ashburner M, Ball CA, Blake JA, Botstein D, Butler H, Cherry JM, Davis AP, Dolinski K, Dwight SS, Eppig JT (2000). Gene ontology: tool for the unification of biology. The Gene Ontology Consortium.. Nat Genet.

[B13] Ryan MM, Huffaker SJ, Webster MJ, Wayland M, Freeman T, Bahn S (2004). Application and optimization of microarray technologies for human postmortem brain studies.. Biol Psychiatry.

[B14] Li J, Gould TD, Yuan P, Manji HK, Chen G (2003). Post-mortem interval effects on the phosphorylation of signaling proteins.. Neuropsychopharmacology.

[B15] Siew LK, Love S, Dawbarn D, Wilcock GK, Allen SJ (2004). Measurement of pre- and post-synaptic proteins in cerebral cortex: effects of post-mortem delay.. J Neurosci Methods.

[B16] Zhai QH, Ruebel K, Thompson GB, Lloyd RV (2003). Androgen receptor expression in C-cells and in medullary thyroid carcinoma.. Endocr Pathol.

[B17] Evans SJ, Choudary PV, Vawter MP, Li J, Meador-Woodruff JH, Lopez JF, Burke SM, Thompson RC, Myers RM, Jones EG (2003). DNA microarray analysis of functionally discrete human brain regions reveals divergent transcriptional profiles.. Neurobiol Dis.

[B18] Khaitovich P, Muetzel B, She X, Lachmann M, Hellmann I, Dietzsch J, Steigele S, Do HH, Weiss G, Enard W (2004). Regional patterns of gene expression in human and chimpanzee brains.. Genome Res.

[B19] Raghavan A, Ogilvie RL, Reilly C, Abelson ML, Raghavan S, Vasdewani J, Krathwohl M, Bohjanen PR (2002). Genome-wide analysis of mRNA decay in resting and activated primary human T lymphocytes.. Nucleic Acids Res.

[B20] Yang E, van Nimwegen E, Zavolan M, Rajewsky N, Schroeder M, Magnasco M, Darnell JE (2003). Decay rates of human mRNAs: correlation with functional characteristics and sequence attributes.. Genome Res.

[B21] Wang Y, Liu CL, Storey JD, Tibshirani RJ, Herschlag D, Brown PO (2002). Precision and functional specificity in mRNA decay.. Proc Natl Acad Sci USA.

[B22] ArrayExpress Database. http://www.ebi.ac.uk/arrayexpress/.

[B23] Bolstad BM, Irizarry RA, Astrand M, Speed TP (2003). A comparison of normalization methods for high density oligonucleotide array data based on variance and bias.. Bioinformatics.

[B24] Ihaka R, Gentleman R (1996). R: A language for data analysis and graphics.. J Comp Graph Stat.

[B25] Significance Analysis of Microarrays. http://www-stat.stanford.edu/~tibs/SAM/.

[B26] Affymetrix. http://www.affymetrix.com.

[B27] LocusLink. ftp://ftp.ncbi.nih.gov/refseq/LocusLink.

[B28] Gene Ontology. http://www.godatabase.org/dev/database/archive.

[B29] GenMapper. http://ducati.izbi.uni-leipzig.de:8080/GenMapper/.

[B30] Do HH, Rahm E (2004). Flexible integration of molecular-biological annotation data: The GenMapper approach.. 9th International Conference on Extending Database Technology: 14-18 June 2004; Heraklion.

